# Encompassing ATP, DNA, insulin, and protein content for quantification and assessment of human pancreatic islets

**DOI:** 10.1007/s10561-017-9659-9

**Published:** 2017-09-15

**Authors:** Meirigeng Qi, Shiela Bilbao, Elena Forouhar, Fouad Kandeel, Ismail H. Al-Abdullah

**Affiliations:** Department of Translational Research and Cellular Therapeutics, Diabetes and Metabolism Research Institute, Beckman Research Institute of City of Hope, 1500 E. Duarte Rd, Duarte, CA 91010 USA

**Keywords:** ATP, DNA, Insulin, Protein, Human islets, Mathematical formula

## Abstract

Islet transplantation has made major progress to treat patients with type 1 diabetes. Islet mass and quality are critically important to ensure successful transplantation. Currently, islet status is evaluated using insulin secretion, oxygen consumption rate, or adenosine triphosphate (ATP) measurement. These parameters are evaluated independently and do not effectively predict islet status post-transplant. Therefore, assessing human pancreatic islets by encompassing ATP, DNA, insulin, and protein content from a single tissue sample would serve as a better predictor for islet status. In this study, a single step procedure for extracting ATP, DNA, insulin, and protein content from human pancreatic islets was described and the biomolecule contents were quantified. Additionally, different mathematical calculations integrating total ATP, DNA, insulin, and protein content were randomly tested under various conditions to predict islet status. The results demonstrated that the ATP assay was efficient and the biomolecules were effectively quantified. Furthermore, the mathematical formula we developed could be optimized to predict islet status. In conclusion, our results indicate a proof-of-concept that a simple logarithmic formula can predict overall islet status for various conditions when total islet ATP, DNA, insulin, and protein content are simultaneously assessed from a single tissue sample.

## Introduction

The pancreas is composed of endocrine and exocrine glands, which play an important role in metabolism and energy homeostasis. The endocrine gland contains the Islets of Langerhans (islets), which consist of insulin-producing β-cells, glucagon-producing α-cells, somatostatin-producing δ-cells, pancreatic polypeptide-producing PP-cells, and ghrelin-producing ε-cells (Bonner-Weir et al. 2015). Islet β-cells secrete insulin to regulate carbohydrate metabolism (Lacy 1967). Type 1 diabetes is an autoimmune disorder that causes destruction of β-cells (Wang et al. 2017; Kracht et al. 2017) and exogenous insulin is often required to treat the disease. However, unphysiological control of glucose homeostasis with insulin in diabetic patients leads to diabetic complications (Cox et al. 1987). Replacement of destructed β-cells by transplanting islets from human cadaveric pancreata achieves euglycemia and insulin independence in subsets of patients with type 1 diabetes (Hering et al. 2016; Ricordi et al. 2016). Thus, assessing the status of islets harvested from the human cadaveric pancreata prior to transplantation is critically important to ensure that the transplantation is effective in controlling patient’s glucose homeostasis (Sweet et al. 2005; Papas et al. 2009).

ATP (Adenosine 5′-triphosphate) molecules, acting as a critical cellular energy source and extracellular messenger, are essential for cell metabolism, signaling pathways, and other biological activities (Zhou et al. 2009; Larsson et al. 2000; Zimmermann 2016). The current method for measuring ATP relies on high-performance liquid chromatography (HPLC) (Manfredi et al. 2002; Chida et al. 2012) and commercially available chemiluminescence kits (Shepherd et al. 2006; Phillippy 1994; Shepherd and Rinker 2004). HPLC is tedious and lengthy, and often associated with failure to quantify ATP content. The commercially available chemiluminescence kits are costly, and the reagents are inefficient to extract total ATP content. Therefore, these approaches for ATP measurement are not sensitive enough to assess islet status. Additionally, the intracellular ATP content of human islets varies largely (Brandhorst et al. 1999). This may be due to inconsistency of donor characteristics (Qi et al. 2015b) and the fact that human pancreas contains various sizes of islets in different proportions (Buchwald et al. 2016). Indeed, ADP/ATP and ATP/DNA ratios have been used to evaluate mitochondrial ATP production for predicting cell or islet condition (Goto et al. 2006; Suszynski et al. 2008). Nevertheless, ATP, DNA, insulin, and protein content of islet preparations have never been integrated into assessments of islet status. In fact, islets have never been free from all other pancreatic tissue making it difficult to specifically predict the characteristic of insulin-producing β-cells. Hence, we hypothesize that analysis of multiple biomolecules, instead of cellular ATP alone, would represent a more robust method to predict the status of human islets. In this study, we first developed a single step procedure for extracting total ATP, DNA, insulin, and protein content. Then, we established a simple, highly sensitive and effective method to measure total ATP content. Lastly, we applied multiple mathematical formulas to integrate values of total ATP, DNA, insulin, and protein content to assess islet status.

## Materials and methods

### Human islet isolation

Human cadaveric pancreata were obtained from local (California) or out-of-state organ procurement organizations. All pancreata used in this study were obtained with research consent from next-to-kin of donors, and the study was approved by the institutional review board of City of Hope. All islet isolations were carried out at a good manufacturing practice (GMP) facility at the City of Hope using standard operating procedures (Qi et al. 2015a). Tissue digestion was carried out using a modified semiautomatic method described previously (Qi et al. 2015a; Ricordi et al. 2016). Digested tissue was purified using a COBE 2991 cell processor (COBE laboratories Inc., Lakewood, CA, USA). The purification was conducted using a continuous gradient by loading 1.100 g/mL and 1.077 g/mL digested tissue (≤40 mL/run) in cold (4 °C) preservation solution. The purified tissue was collected into 7 fractions. Islet purity was assessed using dithizone (DTZ), and the fractions were pooled according to their purity and estimated tissue volume. The islets were washed and cultured immediately after the isolation process (Qi et al. 2015a). Islets were cultured in Connaught Medical Research Laboratories (CMRL)-1066 supplemented media (pH 7.4) with 0.5% human serum albumin (Baxter Healthcare Corporation, Irvine, CA, USA) and 0.1 μg/mL insulin-like growth factor-1 (Cell Sciences, Canton, MA, USA). Islets that were >80% pure and >90% viable were used in this study. Human islets were counted using DTZ staining, and the islet equivalent (IEQ) number was extrapolated as described previously (Ricordi et al. 1990).

### Extraction of total ATP, DNA, insulin, and protein content

Islet aliquots were washed twice with cold DPBS (MediaTech, Inc. Manassas, VA, USA) and centrifuged twice for 1 min at 100×*g* (Eppendorf Centrifuge 5415D, Eppendorf, Westbury, NY). At the end of centrifugation, supernatant was removed and 600 µL of Somatic cell ATP releasing reagent (ARA) (Sigma-Aldrich, St. Louis, MO, USA) was added to the islet pellets before the samples were sonicated on ice for 1 min (Branson Sonifier 250, Danbury, CT). After sonication, 400 µL of ARA was added to the sample, and the sample was centrifuged for 15 min at 1400×*g* (4°C). Lastly, 800 µL of supernatant was collected for further measurement of total ATP, DNA, insulin, and protein content.

### Evaluation of highly sensitive bioluminescent ATP assay

Total ATP content was quantified using an assay developed in our laboratory at City of Hope. ATP is hydrolyzed to AMP and pyrophosphate when luciferase catalyzes luciferin to oxyluciferin, during which light is produced and detected. Total ATP was associated to relative light units (RLU), which was measured using a Revelation MLX Version 4.06 Microtiter Plate Luminometer (Dynex Technologies, Inc., Chantilly, VA). The standard curve between ATP (Fisher BioReagents, Pittsburgh, PA) concentrations (0, 97, 195, 390, 790, 1563, and 3125 nM) and RLU was established. An ATP standard curve was established for each assay performed using a single stock solution of 0.1 M ATP diluted in lysis buffer. Lysis buffer (1% lecithin, 25 mM Tris-PO4, 2% CHAPS, 15% glycerol and 1% BSA) was filtered and stored at 4 °C until used. Briefly, 40 µL of the standard or samples was loaded in duplicate in a 96-well plate (COSTAR 3915 FT bottom). Subsequently, 90 µL of freshly prepared luciferase buffer (3.2635 mM magnesium sulfate, 24.339 mM Tricine, 1.3054 mM Magnesium carbonate, 0.122 mM EDTA, 6.1 mM DL-Dithiothreitol (DTT) (Sigma-Aldrich), 0.183 mg/mL coenzyme A (Avanti Polar Lipids, Alabaster, Alabama), 0.611 mM luciferin (d-Luciferin Firefly) (Photinus pyralis, Biosynth, Switzerland), and 0.11 ng/mL Luciferase (Roche Applied Science, Mannheim, Germany) was added. Luciferase was added to luciferase buffer immediately before reading the plate. As needed, the concentration of luciferase was increased five or tenfold to increase the sensitivity of the assay. Luminescence was read using a TECAN Magellan reader, and the standard curve was established. ATP concentrations of the measured samples were extrapolated from the ATP standard curve.

### DNA content measurement

Total DNA content was measured using a Quant-iT PicoGreen dsDNA Assay kit (Invitrogen, Carlsbad, CA, USA) following manufacturer instructions. DNA standards ranged from 10 to 1000 ng/mL. Concentrated human islet samples were diluted in 1X TE (Tris–EDTA). Samples and standards were plated in 96-black-well plates (Corning 3915, Corning Inc., Corning, NY) and read (excitation 485 nm, emission 535 nm) using a Tecan Magellan V 6.5 Genios (Tecan Systems, Inc., San Jose, CA). Sample concentrations were determined from the standard curve.

### Insulin content measurement

Insulin ELISA kit (Mercodia, Uppsala, Sweden) was used to measure total insulin content following manufacturer instructions. Plates were read using the Tecan Magellan V 6.5 Genios at absorbance of 450 nm. Sample concentrations were determined from the standard curve.

### Protein content measurement

BCA Protein Assay Kit (Thermo Scientific Pierce, Rockford, IL, USA) was used to measure total protein content using the microplate procedure. 1X PBS (MediaTech, Inc., Manassas, VA) was used as the diluent for the standards and was also used to dilute concentrated samples ≥2000 µg/mL, when necessary. Bovine serum albumin standards (BSA) ranged from 25 to 2000 µg/mL using the standard protocol. For samples with low protein content, BSA standards ranged from 5 to 250 µg/mL, and the enhanced protocol was used. Samples and standards were plated in 96-well-clear plates (Corning 3590, Corning Inc.) and read by a Tecan Magellan V 6.5 Genios (595 nm absorbance). The concentrations of measured samples were extrapolated from the standard curve using the slope-intercept form.

### Human islet assessment

Human islets were cultured for 24–72 h before use. Aliquots of 100, 200, 400, and 800 IEQ human islets were used to study the correlation between different extraction conditions and total ATP, DNA, insulin, and protein content. Multiple mathematic formulas incorporating total ATP, DNA, insulin, and protein content were randomly tested using data obtained from different islet aliquots to verify the correlation effect. Furthermore, islets were compared under three conditions in terms of total ATP, DNA, insulin, protein content, as well as the calculated value using the optimized formula. The three conditions were: (1) ARA group (n = 10): islets were directly exposed to ARA to extract ATP; (2) 37°C culture group (n = 4): islets were cultured under normal culture condition in CMRL-1066 media at 37°C/5% CO_2_ for 12 h; (3) −80°C freezing group (n = 4): islets were snap frozen at −80°C for 12 h. At the end of experimental conditions (2, 3), islets were collected and washed twice with cold PBS before ARA was added. 200 IEQ islets were used in each condition and the three groups of islets were processed for extraction of total ATP, DNA, insulin, and protein content.

### Statistical analysis

GraphPad Prism (GraphPad Software 6.0, La Jolla, CA, USA) was used to analyze data and generate figures. Linear regression analysis was used to determine the degree of correlation between the islet doses and total ATP, DNA, insulin, protein content, and formula calculated values. One-way analysis of variance (ANOVA) was used to compare among the three islet groups, followed by Tukey’s multiple comparisons test to compare the mean values between any two groups. Values were expressed as mean ± standard error of mean (SEM), *p* < 0.05 was considered significant.

## Results and discussion

Islet β cells are metabolically active and ATP molecules are the source of energy for insulin synthesis and secretion (Henquin et al. 1985, 2006). When glucose is metabolized in β cells, it leads to ATP production, which in turn closes ATP-dependent K^+^ channels, depolarizes the cell membrane, and opens voltage-dependent Ca^2+^ channels. The opening of Ca^2+^ channels triggers extracellular Ca^2+^ influx, causing insulin to be released from β cells (Lumelsky et al. 2001; Felix-Martinez and Godinez-Fernandez 2014; Wills et al. 2016; Luciani et al. 2007). Therefore, assessing ATP alone without insulin is a poor predictor of islet function.

**Fig. 1:**
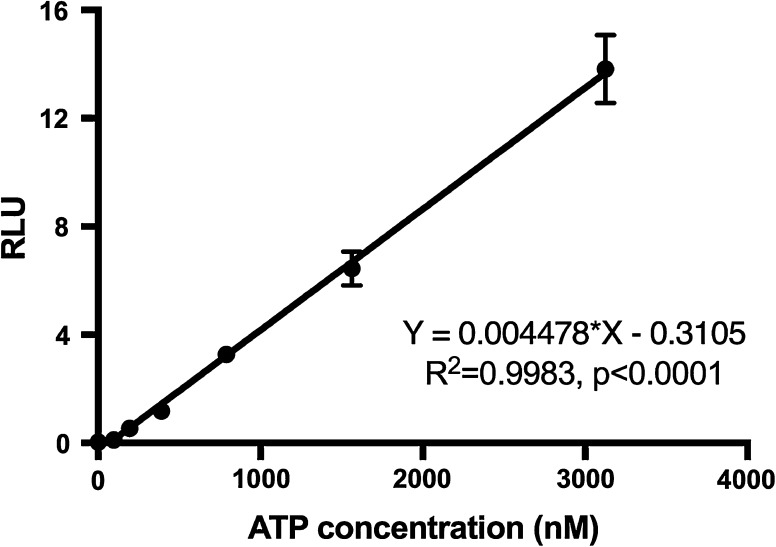
ATP standard curve. The data shows the high correlation (R^2^ = 0.9983, *p* < 0.0001) between standard ATP samples (0, 97, 195, 390, 790, 1563, and 3125 nM) and the RLU reading (n = 17)

In this study, a single-step extraction of total cellular ATP, DNA, insulin, and protein content from human islet samples were evaluated. The extraction process is simple, fast, and can be adapted to any cell type. The biomolecules were quantified and the values were integrated into multiple simplified mathematical formulas to predict islet status. Initially, an ATP standard curve was established to measure total ATP content from islets (Fig. 1). The standard curve was highly correlated (R^2^ = 0.9983, *p* < 0.0001) between standard ATP concentrations and RLU readings (n = 17).

**Fig. 2:**
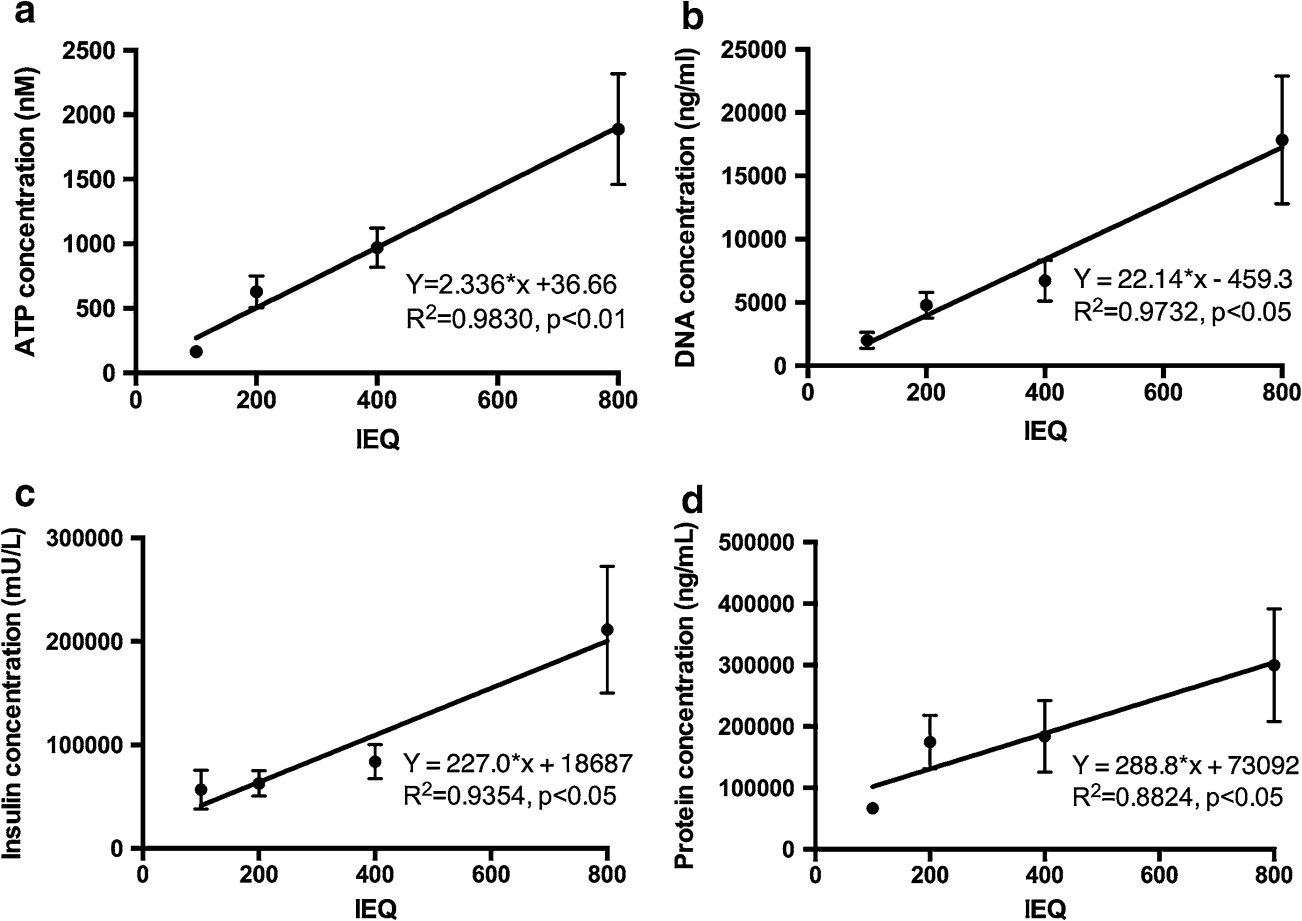
Total ATP, DNA, insulin, and protein contents from different human islets. Measured total ATP, DNA, insulin, and protein contents correlated well with IEQ (100 IEQ, n = 2; 200 IEQ, n = 13; 400 IEQ, n = 10; and 800 IEQ, n = 6). ATP: R^2^ = 0.9830, *p* < 0.01 (**a**). DNA: R^2^ = 0.9732, *p* < 0.05 (**b**). Insulin: R^2^ = 0.9354, *p* < 0.05 (**c**). Protein: R^2^ = 0.8824, *p* < 0.05 (**d**)

After standardizing the ATP measurement, we measured total DNA, insulin, and protein. Figure 2a shows a close association between ATP concentrations and islet IEQ numbers (R^2^ = 0.9830, *p* < 0.01). Total DNA (Fig. 2b, R^2^ = 0.9732, *p* < 0.05), insulin (Fig. 2c, R^2^ = 0.9354, *p* < 0.05), and protein (Fig. 2d, R^2^ = 0.8824, *p* < 0.05) were strongly correlated with an increasing IEQ number (Fig. 2b–d).

**Fig. 3:**
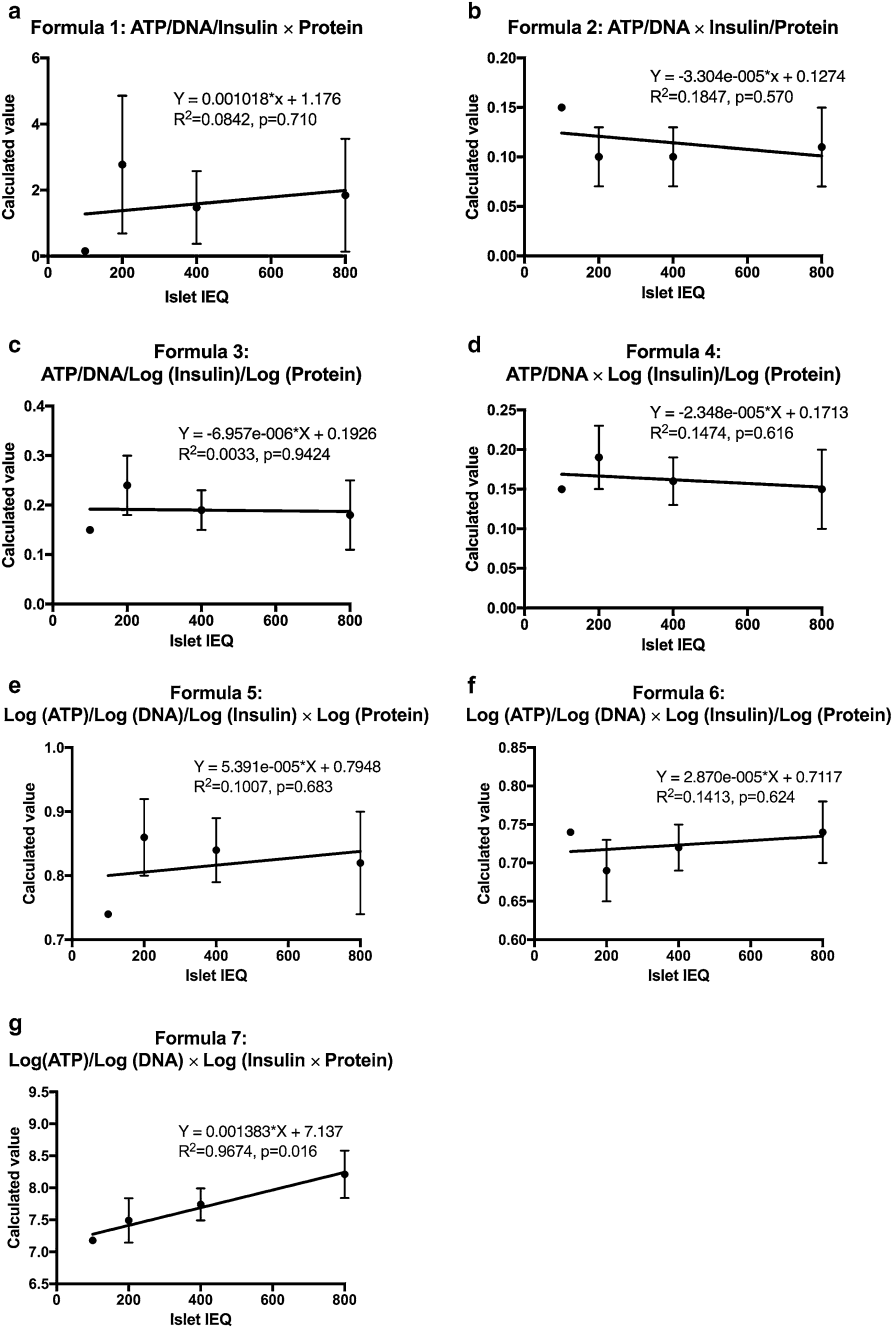
Mathematical formulations integrating total ATP, DNA, insulin, and protein. Seven different mathematical formulations were used to assess human islets using different IEQs: 100 (n = 2), 200 (n = 13), 400 (n = 10), and 800 IEQ (n = 6). Only Formula 7 displayed strong correlation between the calculated values and different number of islets (Fig. 3g, R^2^ = 0.9674, *p* = 0.016). This formula was found to be the best mathematical equation to assess human islets because it integrated all measured parameters: ATP, DNA, insulin and protein

A total of 12 mathematic formulas were applied and tested randomly for evaluating the multiple cellular biomolecules described in different conditions and islet numbers (100, 200, 400, and 800 IEQ). Seven relevant formulas were listed (Fig. 3):

1 $${\text{ATP}}/{\text{DNA}}/{\text{Insulin}} \times {\text{Protein}}$$

2 $${\text{ATP}}/{\text{DNA}} \times {\text{Insulin}}/{\text{Protein}}$$

3 $${\text{ATP}}/{\text{DNA}}/\log ({\text{Insulin}})/\log ({\text{Protein}})$$

4 $${\text{ATP}}/{\text{DNA}} \times \log ({\text{Insulin}})/\log ({\text{Protein}})$$

5 $$\log ({\text{ATP}})/\log ({\text{DNA}})/\log ({\text{Insulin}}) \times \log ({\text{Protein}})$$

6 $$\log ({\text{ATP}})/\log ({\text{DNA}}) \times \log ({\text{Insulin}})/\log ({\text{Protein}})$$

7 $$\log ({\text{ATP}})/\log ({\text{DNA}}) \times \log ({\text{Insulin}} \times {\text{Protein}})$$

Serendipitously, Formula 7 was found to be the most accurate equation verifying the correlation between the 4 biomarkers under different biological conditions tested (Fig. 3g, R^2^ = 0.9674, *p* = 0.016).

**Fig. 4:**
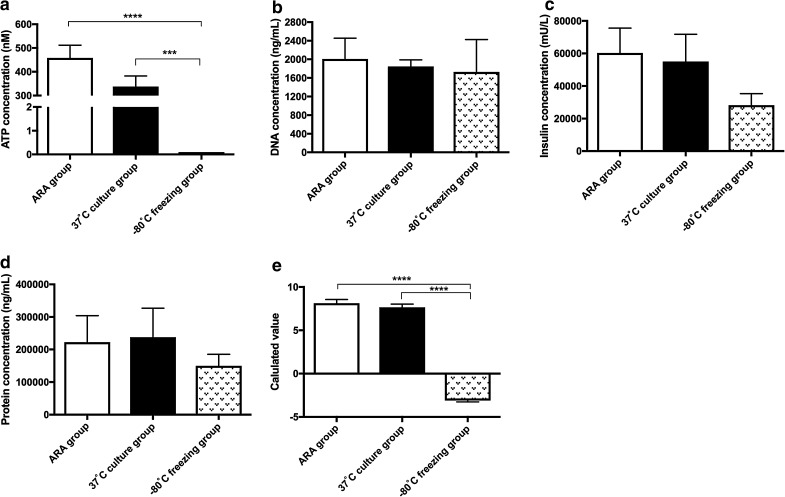
The measured ATP, DNA, insulin, protein, and calculated value of Formula 7 for human islets in ARA, 37°C culture, and −80°C freezing groups. ATP level of frozen islets was 0.10 ± 0.00 nM (the minimal detectable value using this method), which was significantly lower than that of both ARA (458.30 ± 53.91 nM, ****p < 0.0001) and 37°C culture (337.40 ± 45.06 nM, ***p < 0.001) groups (**a**). No significant differences were found for total DNA, insulin, and protein (**b**, **c**, **d**). The Formula 7 calculated value of the −80°C freezing group (−3.10 ± 0.17) was significantly lower than that of both ARA (8.13 ± 0.43, ****p < 0.0001) and 37°C culture (7.65 ± 0.37, ****p < 0.0001) groups (**e**)

Furthermore, we measured total ATP, DNA, insulin, and protein content of human islets under different storage and culture conditions (Fig. 4a–d). The ATP content measured from frozen islets was 0.10 nM (the minimal detectable value using this method), which was significantly lower than ATP content measured from both ATP releasing agent (ARA) (458.30 ± 53.91 nM, *p* < 0.0001) and 37°C culture (337.40 ± 45.06 nM, *p* < 0.001) groups. With regard to total DNA, insulin, and protein levels, we observed no significant differences between frozen islet group and either the ARA or 37°C culture groups. Therefore, Formula 7 was used to evaluate the characteristic of the islets under different conditions (Fig. 4e). The calculated value of the −80°C freezing group (−3.10 ± 0.17) was significantly lower than that of both the ARA (8.13 ± 0.43, *p* < 0.0001) and 37°C culture (7.65 ± 0.37, *p* < 0.0001) groups.

The results of our current study reveal several advantages over current practices:

First, our assessment requires only a single ATP releasing agent to simultaneously extract total ATP, DNA, insulin, and protein content. A previous study showed that ATP could be extracted from living cells using boiling water (Yang et al. 2002). However, variability of extracellular ATP content has been reported, and previous work did not account for DNA, insulin, and protein content (Brandhorst et al. 1999). In this study, we found no difference in calculated ATP, DNA, insulin, and protein content between the ARA and 37°C culture groups, suggesting that our test can be performed immediately after islet isolation. Notably, the ATP level of islets directly exposed to ARA was similar to that of islets after culture, which indicates that the ATP extracting reagent released ATP efficiently and without damaging the ATP molecules.

Second, together with DNA, insulin, and protein, the ATP content could provide comprehensive characteristic of islets represented by a simple mathematical formulation. Previous study reported that ATP and protein levels measured to predict islet transplantation outcome in a pig-to-mouse transplant model (Kim et al. 2009). The intra-islet ATP content was normalized using the porcine islet protein level. However, handpicked islets (100–150 μm in size) were used in the experiment, which does not truly represent islet samples in the clinical setting and therefore limited its application. Several studies used the ADP/ATP or ATP/ADP ratio to predict islet quality, but the results remain controversial (Goto et al. 2006; Sweet et al. 2004). This is partially due to the rapid depletion of ATP and ADP in dead cells; this can lead to high or low ADP/ATP ratios that do not accurately reflect the biological condition of islets. In fact, one study suggested using the combination of ADP/ATP and islet tissue factor measurements as a test for islet potency (Goto et al. 2006). In another report, ATP/DNA, rather than ADP/ATP ratio, was claimed to be a superior indictor of islet viability (Suszynski et al. 2008). Our study improves on the prior literature by integrating not only ATP and DNA content, but also insulin and protein content into a single mathematical calculation. Islet preparation has never been free from acinar tissue and therefore DNA content per se would not indicate specificity to the islet. Therefore, using insulin and total protein, in addition to total ATP and DNA, is of paramount importance to assess insulin-producing β cells (Formula 7).

Lastly, it is well understood that cells cease energy production under freezing conditions (Graumann and Marahiel 1996; Amato and Christner 2009). This was clearly demonstrated in the current study where islets undergoing freezing at −80°C had undetectable levels of ATP content, reflecting the static metabolic condition of the cells. In contrast, islets processed immediately after culture or directly exposed to ARA showed noticeably higher ATP content. These differences were predicted by our mathematical formula (Formula 7) that accurately yielded assessments of different islet characteristics; this suggests that Formula 7 might help unravel the effect of secretagogues to stimulate islets for ATP and insulin upregulation and secretion.

In conclusion, total ATP, DNA, insulin, and protein content can be extracted from human islets using a single step procedure and further quantified. When all the data is integrated into a simple logarithmic formulation, it can predict islet status at different conditions.
